# Genotypic and phenotypic diversity of *Mycobacterium tuberculosis* complex genotypes prevalent in West Africa

**DOI:** 10.1371/journal.pone.0255433

**Published:** 2021-08-26

**Authors:** Stephen Osei-Wusu, Isaac Darko Otchere, Portia Morgan, Abdul Basit Musah, Ishaque Mintah Siam, Diana Asandem, Theophilus Afum, Prince Asare, Adwoa Asante-Poku, Kwadwo Asamoah Kusi, Sebastien Gagneux, Dorothy Yeboah-Manu

**Affiliations:** 1 Noguchi Memorial Institute for Medical Research, University of Ghana, Legon, Ghana; 2 West African Centre for Cell Biology of Infectious Pathogens, University of Ghana, Legon, Ghana; 3 Swiss Tropical and Public Health Institute, Basel, Switzerland; 4 University of Basel, Basel, Switzerland; The University of Georgia, UNITED STATES

## Abstract

Findings from previous comparative genomics studies of the *Mycobacterium tuberculosis* complex (MTBC) suggest genomic variation among the genotypes may have phenotypic implications. We investigated the diversity in the phenotypic profiles of the main prevalent MTBC genotypes in West Africa. Thirty-six whole genome sequenced drug susceptible MTBC isolates belonging to lineages 4, 5 and 6 were included in this study. The isolates were phenotypically characterized for urease activity, tween hydrolysis, Thiophen-2-Carboxylic Acid Hydrazide (TCH) susceptibility, nitric oxide production, and growth rate in both liquid (7H9) and solid media (7H11 and Löwenstein–Jensen (L-J)). Lineage 4 isolates showed the highest growth rate in both liquid (p = 0.0003) and on solid (L-J) media supplemented with glycerol (p<0.001) or pyruvate (p = 0.005). L6 isolates optimally utilized pyruvate compared to glycerol (p<0.001), whereas L5 isolates grew similarly on both media (p = 0.05). Lineage 4 isolates showed the lowest average time to positivity (TTP) (p = 0.01; Average TTP: L4 = 15days, L5 = 16.7days, L6 = 29.7days) and the highest logCFU/mL (p = 0.04; average logCFU/mL L4 = 5.9, L5 = 5.0, L6 = 4.4) on 7H11 supplemented with glycerol, but there was no significant difference in growth on 7H11 supplemented with pyruvate (p = 0.23). The highest release of nitrite was recorded for L5 isolates, followed by L4 and L6 isolates. However, the reverse was observed in the urease activity for the lineages. All isolates tested were resistant to TCH except for one L6 isolate. Comparative genomic analyses revealed several mutations that might explain the diverse phenotypic profiles of these isolates. Our findings showed significant phenotypic diversity among the MTBC lineages used for this study.

## Introduction

Tuberculosis (TB) remains a global health burden and it is the leading cause of human death from a single infectious agent [1]. TB is caused by members of the *Mycobacterium tuberculosis* complex (MTBC) which is made up of about 11 genetically highly related subspecies or ecotypes [2]. Human TB is caused mainly by *M*. *africanum* and *M*. *tuberculosis*; subdivided into nine main phylogenetic lineages (L) which are further regrouped into ‘modern’ and ‘ancient’ lineages [3–8] and exhibit a phylogeographical structure.

A number of studies have now demonstrated the effects of strain diversity among bacterial pathogens [9–13]. These studies highlight the fact that certain strains may cause more invasive diseases than others due to differences in the expression of virulence factors either encoded chromosomally or carried on transmissible elements [14,15]. However, examples of horizontal gene transfer which leads to acquisition of classical pathogenicity islands have not been reported in MTBC, although the MTBC lineages and strains differ phenotypically [13,16,17]. There is now experimental evidence for strain phenotypic diversity in the transmissibility and as well as in the immune response and clinical presentation [18–20]. Some MTBC strains have been associated with large outbreaks, and a propensity to develop drug resistance [21–23]. These observations confirm the need to better understand the diversity in the pathogenesis of the different lineages of MTBC.

Advances in genomics such as whole genome sequencing (WGS) have provided a better understanding of MTBC pathogenesis which includes the identification of essential virulent genes involved in cell surface proteins [24], key enzymes and proteins involved in signal transduction systems [25]. Comparative genomic analyses using WGS have led to the identification of a number of non-synonymous mutations within some of these genes. Based on the observed genomic diversity, some lineages were suspected to be more virulent than others [26,27]. Phenotypic diversity within the MTBC may be attributed to presence of pseudo-genes [28], impaired secretion of virulence-associated proteins [29], and the number of disrupted genes involved in bacterial carbohydrate, lipid and micronutrient metabolism [7,30,31]. However, not all mutations may be translated to phenotypic diversity due to redundancy in specific MTBC pathways [32,33]. Hence, there is a need to investigate the implications of these genomic diversity on the phenotypes expressed by the different lineages.

Most studies on the genotypic and phenotypic characterization of MTBC have concentrated on Mtb lineages with few recent studies on Maf lineages [6,34–37]. However, analysis of the prevalence and spatial distribution of MTBC in Ghana shows that L5 is the most dominant lineage after Lineage 4 (L4) [38]. Ghana is one of the few countries in the world with six out of the 9 phylogenetic lineages of the MTBC causing TB in appreciable numbers and proportions [38]. This makes Ghana a good place to comparatively study the effect of the genetic diversity of the three main MTBC lineages circulating in West Africa (L4 of Mtb and L5 & L6 of Maf) and their phenotypic implications [39]. This study therefore investigated the diversity in the phenotypic profiles of the main prevalent genotypes in West Africa.

## Materials and methods

### Ethical consideration

Ethical clearance was obtained from the Institutional Review Board (IRB) of Noguchi Memorial Institute for Medical Research (NMIMR), University of Ghana (Federal wide assurance number: FWA00001824) and the Ethics Review Committee of the Korle-Bu Teaching Hospital. The details of the study were explained to each participant and written informed consent was obtained from all participants before voluntary enrolment. For participants below 18 years, child assent was sought from the participants and consent from their parents or guardians.

### Mycobacterial isolates

Isolates used in this study comprised of the three prevalent MTBC lineages in Ghana and West Africa (L4 of Mtb and L5 & L6 of Maf). In total, thirty-six (36) distinct spoligotypes were selected from the Cameroon and Ghana sub-lineages of L4 as well as sub-lineages of L5 and L6 as shown in Table 1. We selected L5 and L6 isolates based on a pre-defined clustering (C) into 5 and three sub-population, respectively [36]. Only isolates susceptible to the two most potent anti-TB drugs, isoniazid and rifampicin and that had been whole genome sequenced [36] were used in this study.

**Table 1:** Mycobacterial isolates (lineages/sub-lineages) used for each experiment.

		Growth on 7H11(TTP&CFU)		Growth on L-J		Growth in Liquid medium		Nitrite		Nitrate		Urease test		Tween hydrolysis		TCH	
Lineage(L)	Sub-lineage (s-L)	s-L	L	s-L	L	s-L	L	s-L	L	s-L	L	s-L	L	s-L	L	s-L	L
**L4**	Cameroon	1	2	3	5	2	6	2	6	5	9	4	8	2	4	5	10
	Ghana	1		2		4		4		4		4		2		5	
**L5**	L5.C1	1	5	2	5	1	8	2	6	2	9	2	9	2	10	2	10
	L5.C2	1		1		2		2		2		2		2		2	
	L5.C3	1				2		2		2		2		2		2	
	L5.C4	1				1				1		2		2		2	
	L5.C5	1		2		2				2		1		2		2	
**L6**	L6.C1	1	3	2	5	1	6	1	4	3	7	2	8	2	6	3	10
	L6.C2	1		2		3		2		3		4		2		4	
	L6.C3	1		1		2		1		1		2		2		3	
**Total number**			**10**		**15**		**20**		**16**		**25**		**25**		**20**		**30**

The table summarizes the total numbers of mycobacterial isolates used for each experiment. It displays the number of each lineage as well as sub-lineage.

s-L = sub-lineage, L = lineage.

All the mycobacterial isolates were obtained from the sputum of individuals who participated in a previous study [38] and cultured as previously described by Yeboah-Manu *et al* [40]. The isolates were confirmed as MTBC by PCR amplification of the Insertion Sequence *6110* (IS*6110*) and genotyped into lineages and sub-lineages using spoligotyping and SNP typing [41]. Susceptibility to isoniazid and rifampicin were determined by the microplate alamar blue cell viability assay [42].

All subsequent phenotypic assays were carried out in duplicates and H37Rv was used as the positive control for the assays.

### Growth assessment

#### Growth assessment on solid media

Growth assessment on solid media was performed on both Löwenstein-Jensen (L-J) and 7H11 Middlebrook agar (7H11). Löwenstein-Jensen media was supplemented with either glycerol or pyruvate to assess the utilization of different sources of carbon. A loop-full of mycobacteria growing at the log phase was homogenised into cell suspension at McFarland 1.0 for all the assays in this study. The cell suspension was inoculated on the L-J media slants in duplicates for the growth assessment on media slants. Growth was observed every week for 5 weeks and recorded as degree of positivity as previously shown [43].

Utilization of carbon source was further assessed on 7H11 media plates. All the 7H11 media plates were supplemented with OADC and in addition supplemented with glycerol (7H11+G) or pyruvate (7H11+P) or both (7H11+G+P) or none (7H11). Homogenized mycobacterial isolates at McFarland 1.0 were diluted in 10-fold and 100μL was cultured on the 7H11 plates in triplicates. The time to positivity (TTP) was recorded and the colony forming unit per mL (CFU/mL) was calculated.

#### Growth assessment in liquid medium

Growth rate was assessed in liquid medium using 7H9 Middlebrook broth (7H9) supplemented with 10% ADC (Difco), 0.05% Tween-80, and 0.2% w/v pyruvate [44]. Approximately 5 mL of the 7H9 medium in 25 mL glass tube were inoculated with 200 μL of the mycobacterial suspensions at McFarland 1.0. We performed regular aeration of the tubes under sterile conditions to ensure optimal growth conditions. Absorbance at OD_600_ was measured daily.

### Nitric oxide assay

Nitric oxide production was determined by measuring the concentrations of nitrate and nitrite released by the different isolates. We determined the quantity of nitrite and nitrate at the log phase at McFarland 1.0 using Nitric oxide assay kit (Invitrogen) as previously described [45]. Briefly, this kit uses the enzyme nitrate reductase to convert nitrate to nitrite. Both nitrate and nitrite were subsequently detected by incubating the reaction mix with a known volume of premixed Griess reagent. Detection was made as a coloured azo dye product of the Griess reaction that absorbs visible light at 540 nm. Concentrations of nitrate and nitrite were extrapolated from a constructed standard curve from standards obtained from the Nitric oxide assay kit.

### Urease activity test

The mycobacterial suspensions at McFarland 1.0 were inoculated into 25 mL glass tubes with 5 mL of urea broth in duplicates. The set-up was incubated at 37°C without CO_2_. The absorbance was measured at an OD of 630 nm at 1, 3 and 7 days. A tube with urea broth only was used as negative control. A colour change in broth from yellow to dark pink or red is indicative of a positive reaction whereas a negative test had no colour change.

### Tween hydrolysis test

Tween hydrolysis reagent was prepared by adding 0.5 mL of Tween 80 and 2 mL of a 0.1% aqueous solution of neutral red to 100 mL of phosphate buffer at pH 7.0. The reagent was then dispensed in amounts of 5 mL into 16 x 125 mm screw-cap tubes and autoclaved at 121°C for 10 minutes. A loop-full of mycobacterial isolates growing at the log phase on 7H11 was inoculated into the screw-cap tubes with the tween hydrolysis reagent at McFarland 1.0 in duplicates. The set-up was incubated at 37°C without CO_2_. The absorbance was measured at an OD of 630 nm at 1, 3, 5 and 7 days. A tube inoculated with *M*. *aurum* was used as positive control and a tube with only tween hydrolysis reagent was used as negative control. A colour change from umber to pink or red was indicative of a positive reaction whilst a negative test retained the umber colour.

### Thiophen-2-carboxylic acid hydrazide (TCH) test

We evaluated the drug susceptibility pattern of the selected mycobacterial strains against low concentration of Thiophen-2-carboxylic acid hydrazide (TCH). Four different batches of Löwenstein-Jensen media were prepared for this experiment. Two batches were dispensed as drug-free control media with glycerol and pyruvate; to the other two with either glycerol or pyruvate, we added sufficient filter-sterilized TCH to make a final concentration of 2 μg/mL. A 10-fold dilution was made in sterile saline from a culture at a log phase and 10^−1^ and 10^−3^ dilutions were cultured on the L-J media. A volume of 0.1 mL of each dilution was inoculated on each batch of L-J media. *Mycobacterium bovis* was used as a positive control. All cultures were incubated for 4 weeks at 37°C and a strain was recorded as resistant to TCH if growth on the drug-containing medium was equal to or greater than 1% of that observed on the drug-free control medium.

### Comparative genomic analysis

Whole genome sequence data of the selected isolates were obtained from the European nucleotide archives (accession numbers tabulated in S1 Table). They were mapped unto the H37Rv reference genome (accession number. NC_000962) [46,47] using customized bash algorithms communicating to BWA, samtools and bcftools after quality checks with fastqc and trimming using Trimmomatic to obtain the whole genome sequence in fasta format. Specific genes of interest (S2 Table) were extracted using an in-house shell script that communicates to EMBOSS [48] and compared to their respective sister genes in H37Rv for identification of mutations.

### Data analysis

Data was analysed and figures were developed by using RStudio Version 1.2.5033 (RStudio, Inc., Boston, MA, USA) with readr, plyr and ggplot2 packages [49–51]. Analysis of Variance (ANOVA) was used to compare the differences across the three lineages of MTBC. In certain circumstances, a two-way ANOVA was used to factor in other variables. *Ad hoc* testing, tukey test was carried out for analysis that showed significance difference. All analyses were carried out with significance level set at p-value less than 0.05 at 95% confidence level.

All the data for the different lineages in the constructed figures were represented by their standard colours: red = L4, brown = L5 and green = L6.

## Results

### Growth rate and carbon utilization

#### Utilization of carbon source on L-J media

**Fig 1:**
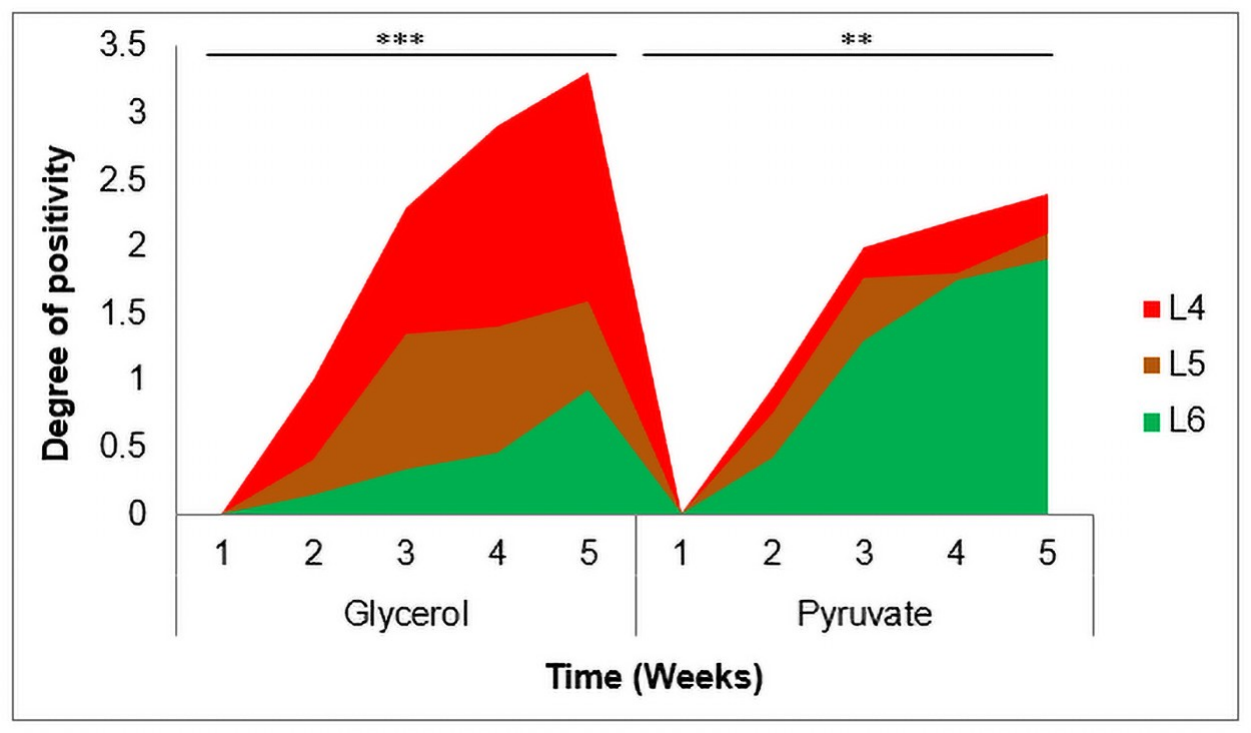
Utilization of different carbon sources on Löwenstein-Jensen media slants. Growth was compared as degree of positivity (WHO standard for grading Mtb growth on L-J) on both glycerol and pyruvate supplemented media slants. Two-way analysis of variance (ANOVA) considering Time as a factor showed significant difference between the 3 lineages on glycerol (p<**0.001**) and on pyruvate (p = **0.005**) supplemented media.

We first assessed the utilization of different carbon sources (glycerol and pyruvate) for isolates representative of the three different lineages (Table 1) on L-J media slants. Growth was compared as degree of positivity on glycerol or pyruvate supplemented media slants (Fig 1). Analysis of the average degree of positivity at each time point showed significant difference in growth pattern of the three lineages on glycerol (p<0.001) and on pyruvate (p = 0.005) supplemented media. Further comparison of growth on pyruvate supplemented L-J media showed difference between L6 and L4 (p = 0.003) but not between L5 and L4 (p = 0.2) and L6 versus L5 (p = 0.2). Growth of L4 on glycerol supplemented L-J media over the 5 weeks of observation was significantly higher compared to that on pyruvate (p = 0.004). However, L6 isolates preferred to utilize pyruvate for growth compared to glycerol (p<0.001). Growth of L5 isolates appeared to be similar on both glycerol and pyruvate supplemented L-J media (p = 0.05). This suggests that L5 isolates utilize both glycerol and pyruvate at the same rate for growth.

### Growth rate on 7H11

#### Time to positivity

**Fig 2:**
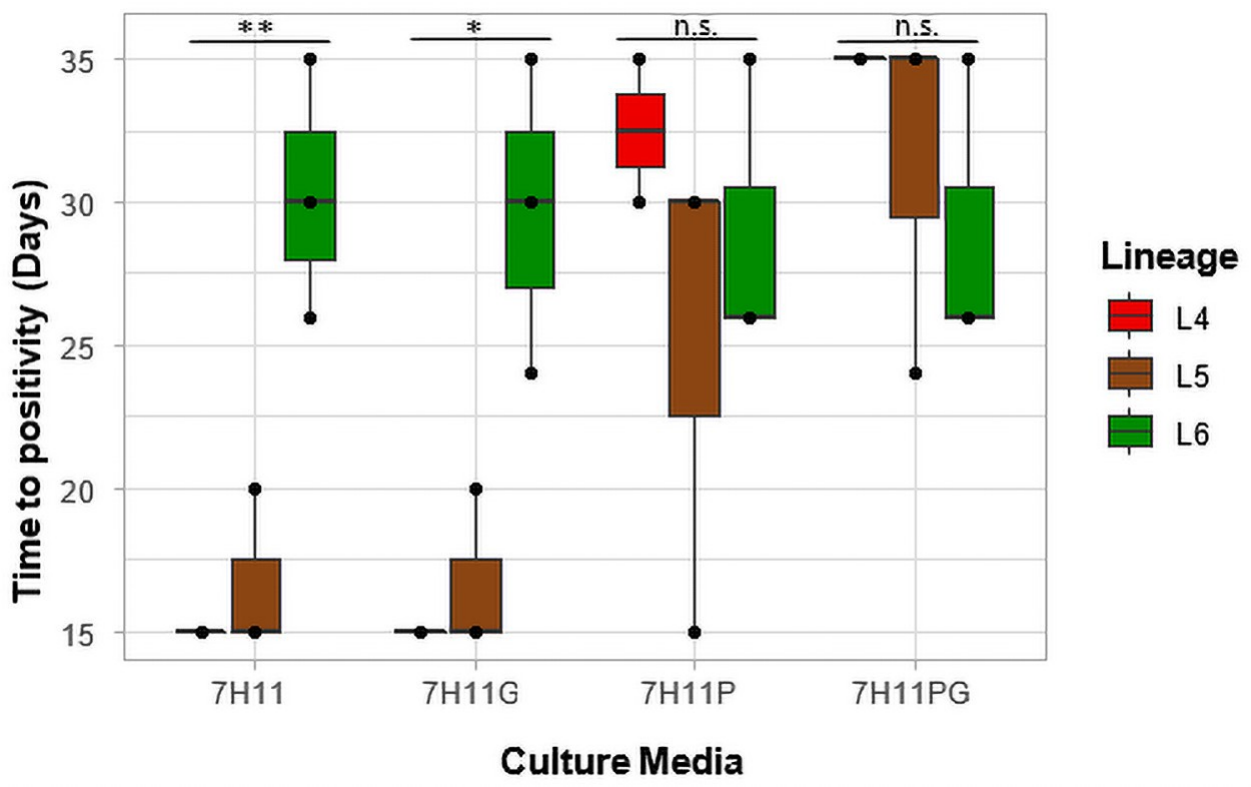
Time to positivity (TTP) on 7H11 media. The average time to positivity was measured in terms of the number of days it took for the first colonies to appear for CFU determination and plotted against the various media compositions.

We assessed the growth of the three lineages on 7H11 media plates. The time to positivity (TTP) was measured in terms of the number of days it took for the first colonies to appear for CFU determination (Fig 2). Lineage 4 isolates had the lowest average time to positivity of 15 days (SD = 0) followed by L5 (Average TTP = 16.7 days, SD = 2.9) and L6 (Average TTP = 30.3 days SD = 4.5). There were statistically significant differences between the time to positivity for the three different lineages on 7H11 only media (overall p-value = 0.006; L5-L4 = 0.86, L6-L4 = 0.01, L6-L5 = 0.01).

There was also significant difference between the average TTP of the lineages (Average TTP: L4 = 15.0 days, SD = 0; L5 = 16.7 days, SD = 2.9; L6 = 29.7 days, SD = 5.5) on 7H11+G media (overall p-value = 0.01; L5-L4 = 0.89, L6-L4 = 0.02, L6-L5 = 0.02). There was however no significant difference between the TTP (Average TTP: L4 = 32.5 days, SD = 3.5; L5 = 25 days, SD = 8.7; L6 = 29 days, SD = 5.2) on 7H11+P media (p = 0.5). The average TTP of the lineages (Average TTP: L4 = 35 days, SD = 0; L5 = 31.3 days, SD = 6.4; L6 = 29 days, SD = 5.2) on 7H11+P+G media was similar to that of 7H11+P (p = 0.5).

#### Determination of CFU

**Fig 3:**
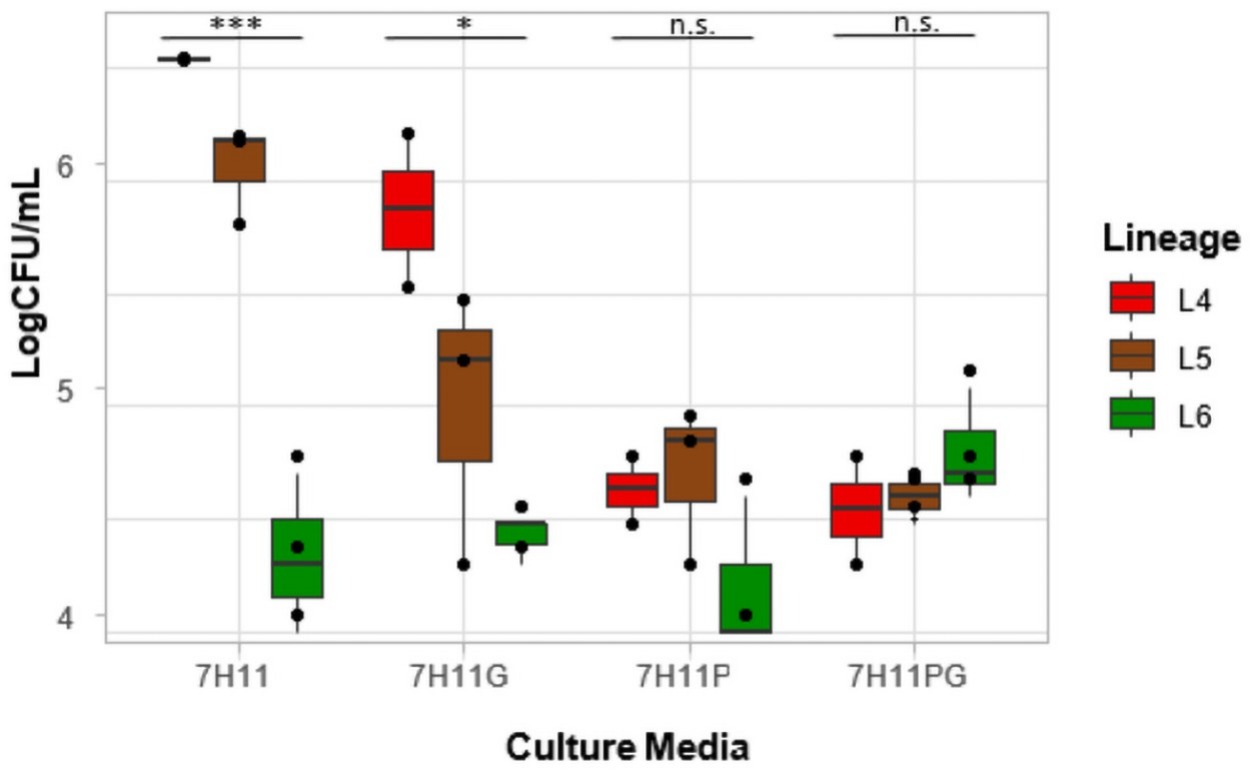
Growth rate on 7H11 media. The average logCFU/ml was plotted against the different supplemented media.

The number of colonies were counted and the CFU per mL was calculated for each mycobacterial isolate. A barplot of logCFU/mL was made for each of the different supplemented media (Fig 3). The logCFU/mL of the lineages was significantly different on 7H11 (overall p-value<0.001; L5-L4 = 0.2, L6-L4<0.001, L6-L5 = 0.001) and 7H11+G media (overall p-value = 0.04; L5-L4 = 0.18, L6-L4 = 0.04, L6-L5 = 0.34). For growth rate on 7H11, L4 had the highest average logCFU/mL of 6.5 (SD = 0.005) followed by L5 with 6.1 (SD = 0.2) and L6 with 4.3 (SD = 0.3). The same trend was observed on 7H11+G media (Average logCFU/mL: L4 = 5.9, SD = 0.5; L5 = 5.0, SD = 0.6; L6 = 4.4, SD = 0.1). There was however no significant difference among the isolates when growing on 7H11+P (p = 0.23) and 7H11+P+G (p = 0.46).

### Growth rate in liquid medium

**Fig 4:**
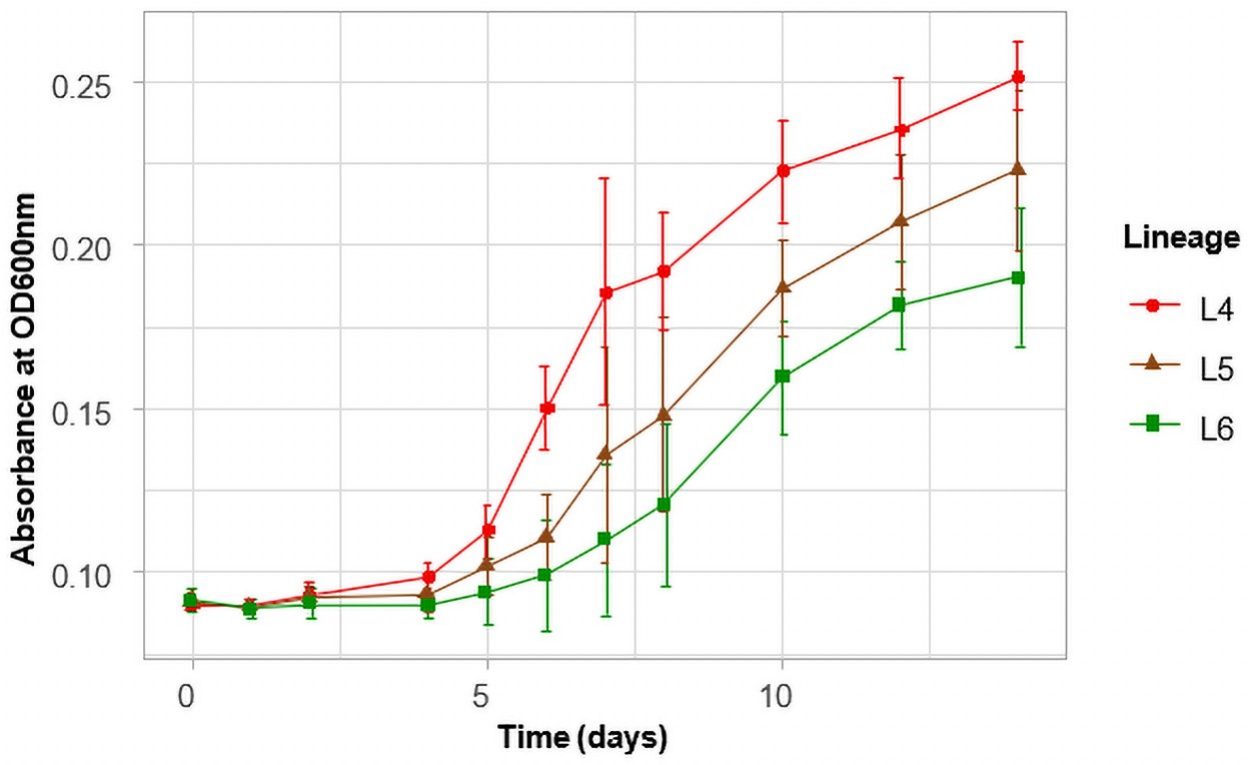
Growth rate in liquid medium. Optical density at 600nm was measured at the data points (days) and indicated by the symbols. The data points are the averages of individual strains belonging to the 3 different lineages.

Since the growth rate of the three lineages on pyruvate supplemented solid media was similar, we compared the growth rate in 7H9 medium with pyruvate as the carbon source. Growth rate was assessed by measuring the optical density at 600nm over a 14-day period at different time points as shown in Fig 4. Again, we showed that there was significant difference in the growth rate of the lineages over the time period of observation (p = 0.0003). Lineage 4 isolates showed the highest average growth rate compared to L5 (p = 0.026) and L6 (p = 0.0002) isolates, respectively. There was, however, no significant difference between isolates of L5 and L6 (p = 0.17). This observation confirms the higher growth rate of L4 isolates compared to the Maf lineages.

### Identification of genotype-specific mutations among genes that may be associated with growth

We compared the amino acid sequence of 22 genes (S2 Table) associated with growth of the MTBC using H37Rv as reference. Three mutations, R16P, Y98H & V75I, in the *ftsE* gene which is associated with cell division were found in L5 and L6 specifically L6.C3, L5.C2 & L5.C1 (Table 2). Also, 2 mutations, R3L and S455A, in the *pstP*, which codes for phosphoserine/threonine phosphatase were found in all L5 and L6 isolates. Again, mutations, A196G and D43A, in *suhB* associated with extragenic suppressor protein, SUHB, were observed in L5 and L6 isolates, respectively. A mutation (E71stop) in *whiB3*, a transcriptional regulatory protein, was found in all L5 isolates. A gene that codes for a probable resuscitation-promoting factor (RPFE), *rpfE*, was also found to have a R126Q mutation in all the isolates and an additional A87V in L5.C1 isolates.

**Table 2:** Mutational analysis of genes that may be associated with growth rate.

Isolate	Gene name	Mutation	Description
L6.C3, L5.C2 & L5.C1	*ftsE*	R16P, Y98H & V75I	Putative cell division ATP-binding protein FTSE (septation component-transport ATP-binding protein ABC transporter)
L6.2 (one isolate:1283)	*hmp*	T125I	Possible Hemoglobine-related protein HMP. Possible ketosteroid-9-alpha-hydroxylase.
L5 only	*pknB*	V251L	Transmembrane Serine/Threonine-protein kinase B PKNB (Protein Kinase B) (STPK B)
Maf only	*pstP*	R3L	Phosphoserine/Threonine Phosphatase PSTP
Maf only	*pstP*	S455A	Phosphoserine/Threonine Phosphatase PSTP
L5.3 (one isolate: 2196)	*pstP*	S475R	Phosphoserine/Threonine Phosphatase PSTP
All except H37Rv	*rpfE*	R126Q	Probable resuscitation-promoting factor RPFE
L5.C1 only	*rpfE*	A87V	Probable resuscitation-promoting factor RPFE
L6 only	*suhB*	D43A	Possible extragenic suppressor protein SUHB
L5 only	*suhB*	A196G	Possible extragenic suppressor protein SUHB
L5 only	*whiB3*	E71stop	Transcriptional regulatory protein WHIB-like WHIB3

### Nitric oxide production and identification of mutations that could be associated with its impaired production

**Fig 5:**
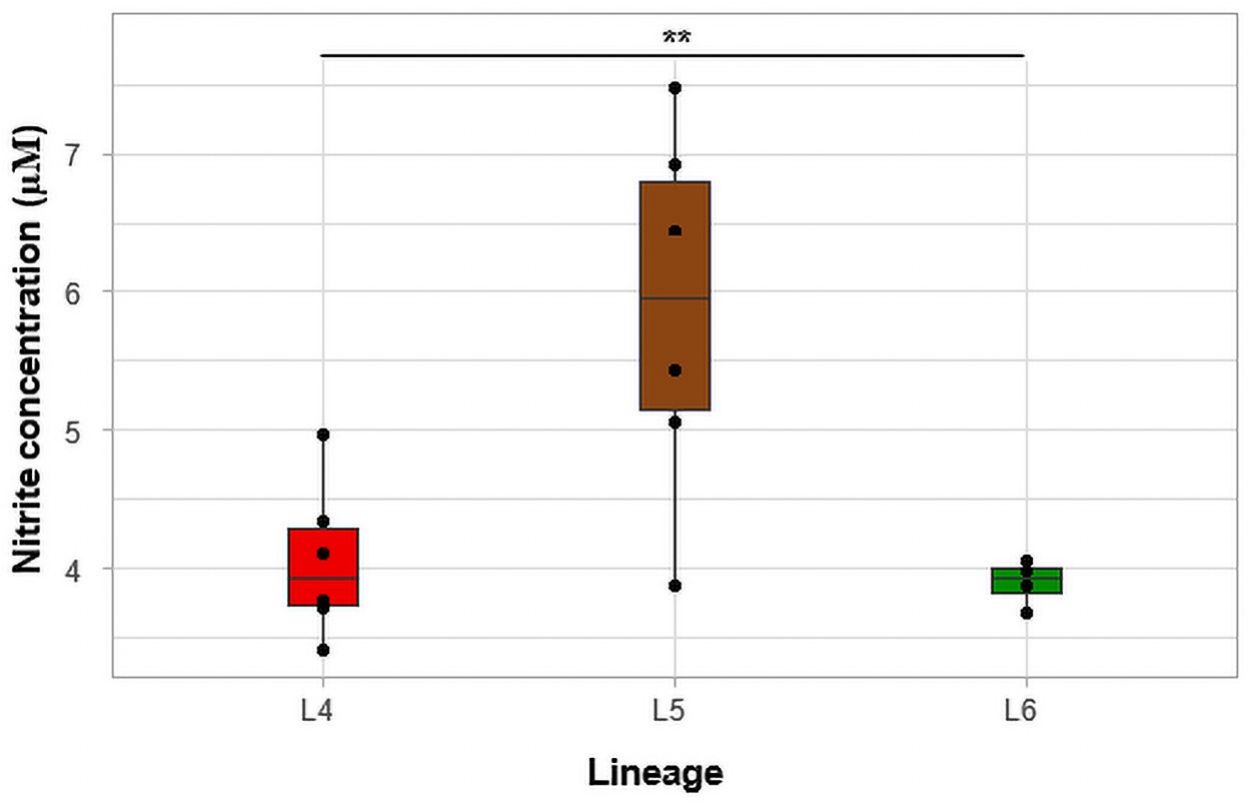
Comparison of nitrite concentration. The individual isolates are represented by the black points in the boxplot.

To evaluate the ability of a lineage to produce nitric oxide, we quantified nitrite and nitrate concentrations in culture supernatant. The nitrite concentrations were determined and mean concentrations extrapolated from a standard curve was plotted per lineage (Fig 5). Lineage 5 isolates had the highest mean concentration of nitrite of 5.87 μM (SD = 1.33) followed by L4 (L5 = 4.06 μM, SD = 0.6) and L6 (L6 = 3.91 μM, SD = 0.2) isolates, respectively. We observed significant difference between the nitrite concentrations of L5-L4 (p = 0.01) and L5-L6 (p = 0.01): however, the mean nitrite concentration of L4 was similar to that of L6 (p = 0.96). Mutational analysis of genes which may be involved in nitrite production of L4 showed mutations, Y389C and V409I in their *narK* transporter genes, *narK1* and *narK4*, respectively (Table 3). A mutation (P18L) in the gene, *narL*, that possibly codes for nitrate/nitrite response transcriptional regulatory protein, NARL, was observed in all L6 isolates.

**Table 3:** Genomic analysis for genes that may be involved in nitric oxide.

Isolate	Gene name	Mutation	Description
L5 only	*narG*	S435R	Probable Respiratory Nitrate Reductase (Alpha Chain) NARG
L6.C1	*narG*	E967G	Probable Respiratory Nitrate Reductase (Alpha Chain) NARG
Ghana (3742)	*narK1*	Y389C	Probable Nitrite Extrusion Protein 1 NARK1 (Nitrite Facilitator 1)
L5.C3 (1186)	*narK3*	A226E	Probable Integral Membrane Nitrite Extrusion Protein NARK3 (Nitrite Facilitator)
Ghana & Cameroon	*narK4*	V409I	Probable Integral Membrane Nitrite Extrusion Protein NARK3 (Nitrite Facilitator)
L6 only	*narL*	P18L	Possible Nitrate/Nitrite Response Transcriptional Regulatory Protein NARL
Cameroon (1175)	*narX*	R49Q	Probable Nitrate Reductase NARX
Maf only	*narX*	D77G	Probable Nitrate Reductase NARX
L6.C1	*Rv0890c*	A355T	Probable Transcriptional Regulatory Protein (Probably LUXR-family)
L6.C3 (2150)	*Rv0890c*	H382Q	Probable Transcriptional Regulatory Protein (Probably LUXR-Family)
Cameroon (1030)	*Rv0890c*	R519H	Probable Transcriptional Regulatory Protein (Probably LUXR-family)
L5.C3 (1186)	*Rv0890c*	E603G	Probable Transcriptional Regulatory Protein (Probably LUXR-family)
L6.C1	*Rv0890c*	V647G	Probable Transcriptional Regulatory Protein (Probably LUXR-family)
All except H37Rv	*Rv0890c*	P866A	Probable Transcriptional Regulatory Protein (Probably LUXR-family)
L5 only	*Rv0890c*	Q286R	Probable Transcriptional Regulatory Protein (Probably LUXR-family)
L6.C2 (1283)	*Rv0890c*	Y300H	Probable Transcriptional Regulatory Protein (Probably LUXR-family)
L5.C2	*Rv1869c*	D265A	Probable Reductase
Cameroon (1175)	*Rv2994*	W214stop	Probable Conserved Integral Membrane Protein

**Fig 6:**
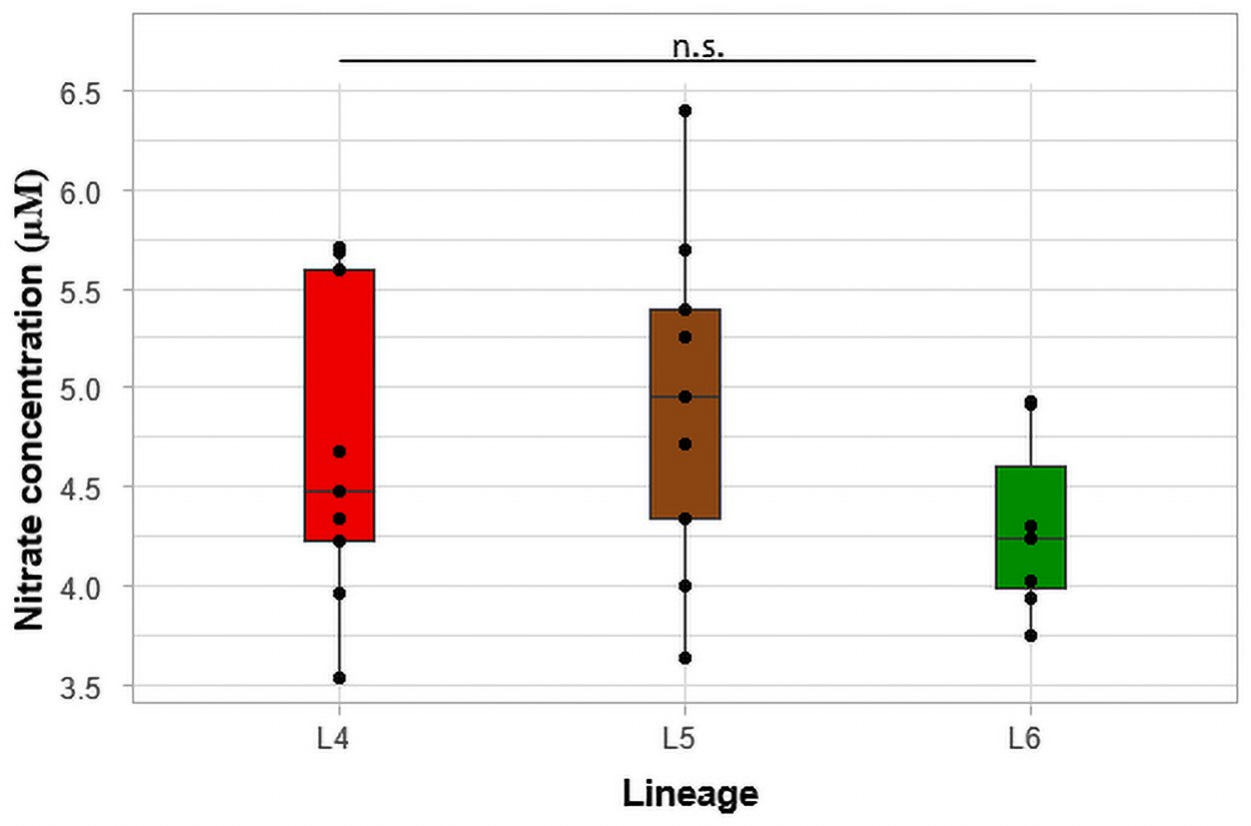
Comparison of nitrate concentration. The individual isolates are represented by the black points in the boxplot.

The nitrate concentrations were determined by converting nitrate to nitrite and the concentration was plotted according to the three lineages (Fig 6). The mean concentrations of nitrate for L4, L5 and L6 isolates were determined as 4.69 μM (SD = 0.8), 4.93 μM (SD = 0.9) and 4.30 μM (SD = 0.5), respectively. Although L5 isolates had the highest mean concentrations of nitrate, followed by L4 and L6, the difference was not statistically significant (p = 0.27). Genomic analysis of the mycobacterial isolates revealed several mutations in genes associated with nitrate reduction. A lineage-specific mutation, P18L, in the *narL* which codes for Nitrate/Nitrite response transcriptional regulatory protein, NARL, was observed among all L6 isolates. Additional *Rv0890c* mutations, A355T & V647G were observed in L6.C1 isolates and Q286R was observed in all L5 isolates (Table 3). We also observed mutations, S435R and E967G, in another nitrate reductase gene, *narG*, in L5 and L6.C1 isolates. Both L5 and L6 showed a common mutation, D77G, in *narX*, which codes for another nitrate reductase, NARX.

### Urease activity test

**Fig 7:**
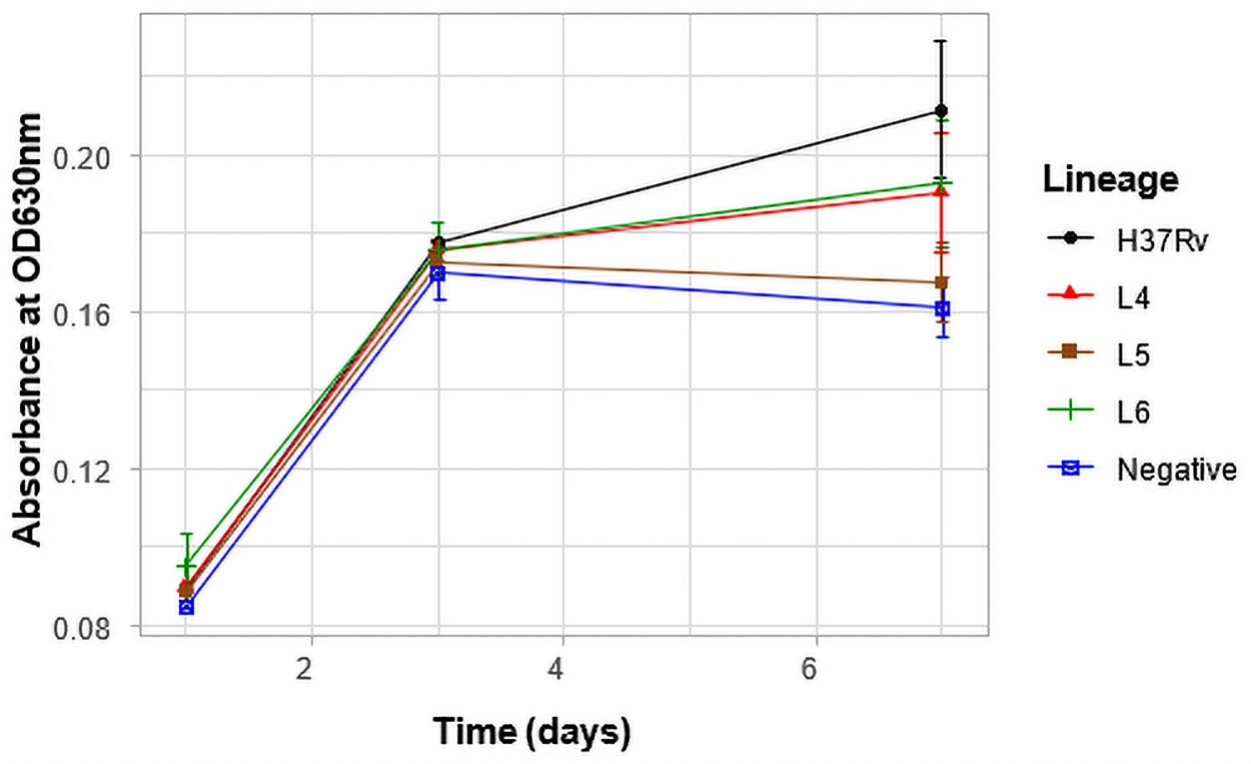
Comparison of urease activity of the three different lineages. A negative control depicted by the blue line was only urea broth. H37Rv was included in the experimental set-up as a positive control (black line).

Urease activity of the three lineages was compared by measuring the absorbance at 630 nm at days 1, 3 and 7 (Fig 7). Each time point represented an average of the representative mycobacterial isolates of the three different lineages (Table 1). This experiment included H37Rv which is known to be positive for urease test as well as the majority of the members of MTBC. The average urease activity of L4 (average urease activity = 0.190, SD = 0.02) and L6 (average urease activity = 0.192, SD = 0.02) isolates was stronger than L5 isolates (average urease activity = 0.167, SD = 0.01). We analyzed the difference in the urease activity at day 7 for the isolates. There was significant difference (p = 0.02) between the average urease activity of the positive control, H37Rv (average urease activity = 0.211, SD = 0.02) and the negative control (average urease activity = 0.161, SD = 0.008). The same trend was observed between H37Rv and L5 (p = 0.02). Two mutations, G98D and Y169C, in the *Rv1395* gene which is associated with a transcriptional regulatory protein were observed in L5.C2 strains (Table 4).

**Table 4:** Genomic analysis for urease activity test.

Lineage	Gene	Mutation	Gene function
L5.C2	*Rv1395*	G98D & Y169C	Transcriptional Regulatory Protein
L5.C1	*mrsA*	T125I	Probable Phospho-Sugar Mutase/Mrsa Protein Homolog

### Tween hydrolysis test

**Fig 8:**
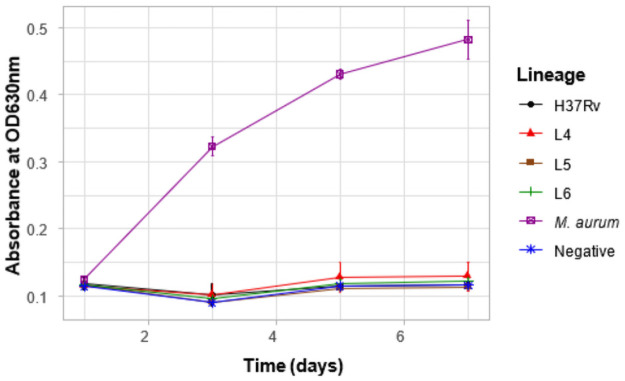
Tween hydrolysis test. A negative control depicted by the blue line had only the Tween hydrolysis reagent. *Mycobacterium aurum* represented by the purple line was included in the experimental set-up as a positive control.

We determined the ability of the mycobacterial isolates to hydrolyze tween by measuring the optical density at 630 nm at days 1, 3, 5 and 7. *Mycobacterium aurum* represented by the purple line was positive whereas the negative control presented by the blue line which is the tween hydrolysis reagent only was negative (Fig 8). All 3 lineages were negative for the tween hydrolysis test. Since all the lineages were negative for tween hydrolysis, mutation analyses for genes that may be involved in Tween hydrolysis were excluded from this study.

### Thiophen-2-carboxylic acid hydrazide (TCH) susceptibility

Susceptibility pattern of the mycobacterial isolates against low concentrations of TCH was determined with a clinical strain of *M*. *bovis* used as a control. All mycobacterial isolates used were resistant to TCH except one strain of L6 (Table 5). Genomic analysis revealed a mutation, A11D, in *furA* gene in L5.C2. There was no additional mutation found in the analysed genes of the isolates susceptible to TCH.

**Table 5:** Thiophen-2-carboxylic acid hydrazide (TCH) susceptibility profile.

Isolate ID	Sub-lineage	Lineage	TCH results	Gene	Mutation	Isolate ID	Sub-lineage	Lineage	TCH results	Gene	Mutation
H37Rv			R	-	-	1283	L6.2	L6	R	-	-
*M*. *bovis*			S	N/A	N/A	1957	L6.2	L6	R	-	-
1036	Cameroon	L4	R	-	-	1102	L6.2	L6	R	-	-
1034	Cameroon	L4	R	-	-	1434	L6.3	L6	R	-	-
1327	Cameroon	L4	R	-	-	2150	L6.3	L6	S	-	-
1307	Cameroon	L4	R	-	-	1289	L6.3	L6	R	-	-
1346	Cameroon	L4	R	-	-	1421	L5.1	L5	R	-	-
1979	Ghana	L4	R	-	-	2910	L5.2	L5	R	*furA*	A11D
2070	Ghana	L4	R	-	-	1010	L5.2	L5	R	*furA*	A11D
1800	Ghana	L4	R	-	-	1786	L5.1	L5	R	-	-
1608	Ghana	L4	R	-	-	2541	L5.3	L5	R	-	-
1448	Ghana	L4	R	-	-	2196	L5.3	L5	R	-	-
1280	L6.1	L6	R	-	-	2384	L5.4	L5	R	-	-
1821	L6.1	L6	R	-	-	1984	L5.4	L5	R	-	-
2016	L6.1	L6	R	-	-	1144	L5.5	L5	R	-	-
1082	L6.2	L6	R	-	-	1313	L5.5	L5	R	-	-

## Discussion

*Mycobacterium africanum* (Maf) is mainly restricted to West Africa for reasons that are not well understood. Previously, studies have delineated distinct phenotypic characteristics such as the association of Maf L5 with ethnicity whereas Maf L6 has been associated with HIV infection and slower progression to active disease [18,39,41,52]. Attempts have been made to further explore these observations with comparative genomics and molecular epidemiology with limited phenotypic studies. Some previous studies involving Maf L6 showed characteristics such as slow growth, preference for pyruvate as a carbon source in a liquid medium and dysgonic growth nature on solid medium [34,44,53,54]. This study therefore, sought to phenotypically characterize L4, L5 and L6 which are the three most dominant MTBC genotypes in West Africa [34,53]. We carried out mutation analyses of the genes involved in the phenotypic assays. However, this study did not carry out complementation experiments to confirm the effects of the observed mutations.

We confirmed the preference of Maf lineages for pyruvate as a carbon source compared to glycerol although L5 also grows fairly well with glycerol (Fig 1). This observation confirms previous comparative genomic studies that revealed the presence of a single nucleotide polymorphism (SNP), E220D, in the *pykA* gene of *M*. *africanum* and *M*. *bovis* which impairs the activity of pyruvate kinase in the metabolism of carbohydrates [44,47,55]. Another SNP, R179S, in the *eno* gene was found to be associated with only L6 which could contribute to the more impaired utilization of glycerol among L6 strains [55].

On the other hand, the observed reduced growth of L4 on pyruvate supplemented media was expected: confirming the loss of ATP for skipping the glycolytic pathway. The difference in growth rate of the 3 lineages on pyruvate was minimal compared to the high disparity observed on glycerol. Hence, for a comparative phenotypic study on L-J slants, it is recommended to use pyruvate as a carbon source to reduce biases. We again observed a higher growth rate for L4 compared to L5 and L6 on 7H11+G and 7H11only media.

The high growth rate of L4 in liquid medium observed in this study is consistent with other studies that reported a longer doubling time or slower growth for Maf from a defined inoculum [33,34,53]. This also confirms the observation made by Castete *et al* when they used biochemical assays to differentiate Maf from Mtb [56]. They also observed a longer time to positivity for Maf. We hypothesize that the slow growth of Maf could be due to mutations in some essential growth genes. In this study, mutations in *ftsE*, *pstP*, *whiB3* and *suhB* genes were detected among the Maf lineages. These genes are associated with cell division and are responsible for control of cell cycle and virulence in bacteria. Thus, amino acid mutations in these genes could lead to defects in cell wall and cell division [57,58]. A future complementation experiments will be needed to validate the effects of these mutations.

Nitric oxide (NO) is a key anti-mycobacterial molecule which plays an important role in the pathogenesis of Mtb [59]. The amount of NO elicited by infected macrophages significantly contributes to the outcome of infection: the less the concentration of NO, the more virulent the pathogen [60]. A study where human macrophages were infected with Mtb under oxygen tension surprisingly showed high concentrations of nitrite from the activity of Mtb instead of the macrophages themselves [61]. The MTBC strains responded to the high concentration of nitrite by stopping their growth. Based on this finding, we hypothesized that; L6 will produce the highest concentration of nitrite/nitrate since it has the lowest growth rate. Interestingly, the reverse was observed for L6 which could be due to a mutation, P18L, in *narL* which codes for nitrate/nitrite response transcriptional regulatory protein, NarL (Table 3). This specific mutation in *narL* has been reported to be associated with L6 strains [62]. According to a KEGG pathway analysis of NarL protein family, impaired NarL expression leads to the impairment of NarGHIJ (nitrate reductase) and ultimately results in reduced nitrite/nitrate concentrations [63].

Nitrogen metabolism of the three lineages was further assessed using urease activity. MTBC urease activity is classified as a virulence factor due to its alkalisation of acidic environment to aid the survival of MTBC [64–66]. Although urease activity is frequently used to differentiate MTBC from other mycobacteria, urea can be used as an alternative source of nitrogen by the MTBC especially in the absence of other nitrogen sources but the levels of utilization may differ depending on the genotypes [67,68]. The highest urease activity was observed in L6 isolates, and this suggests L6 prefers to utilize the alternative pathway by converting urea to carbon dioxide and ammonia [69] instead of nitric oxide production. From comparative mutational analysis of genes associated with urease activity, we identified 3 mutations affecting 2 genes (Table 4). However, the mutations were found among only L5 strains which may explain the limited urease activity of L5 compared to the other genotypes.

Lipids form an important component of the life cycle of MTBC and hence, lipases and esterases are crucial for its pathogenesis [70]. The MTBC lipases hydrolyze host cell lipids into fatty acids which they use as energy source and building material for replication [71,72]. Tween hydrolysis test was carried out to characterize the hydrolytic properties of lipases/esterases of the three different lineages. All three lineages were negative for the tween hydrolysis test as expected for MTBC strains. This observation suggests the use of Tween 80 in Mycobacterial cultures not exceeding 20 days will likely not interfere with MTBC metabolism but rather serve its primary purpose as a non-ionic surfactant [73,74].

Lastly, the ability of mycobacteria to grow in the presence of inhibitory substances such as TCH was used to determine the diversity in virulence. Only one L6 strain was susceptible to TCH which is quite interesting because L6 are phylogenetically more closely related to *M*. *bovis* than other human adapted MTBC lineages [39].

## Conclusion

In conclusion, L4 grows faster than Maf (L5 & L6) and even between the two Maf lineages; L5 grows faster than L6. The other assays carried out in this study also confirm the phenotypic diversity in the three lineages. There was generally significant diversity in the genotypic and phenotypic profiles of the MTBC lineages and the observed phenotypic variations might be explained by mutations identified by the comparative genomic analyses carried out. However, complementation studies need to be carried out to appropriately correlate the observed mutations with the different phenotypic characteristics. This study confirms the earlier reported genomic variations among the lineages which may potentially have implications on microbial physiology. The diversity in pathogen physiology can be used to explain the clinical and epidemiological characteristics of Maf.

## Supporting information

S1 Table Accession numbers for the analysed genes. (XLSX)

S2 Table List of genes used for the study. (XLSX)
